# Towards a taxonomic revision of the lycophyte genus *Selaginella* (Selaginellaceae) in Guizhou, China: new species, new records and cryptic species

**DOI:** 10.3897/phytokeys.274.184803

**Published:** 2026-04-29

**Authors:** Jun-Kai Huang, Zhi-You Guo, Chao-Yi Deng, Shi-Yong Dong

**Affiliations:** 1 Key Laboratory of National Forestry and Grassland Administration on Plant Conservation and Utilization in Southern China, South China Botanical Garden, Chinese Academy of Sciences, Guangzhou 510650, China South China Botanical Garden, Chinese Academy of Sciences Guangzhou China; 2 University of Chinese Academy of Sciences, Beijing 100049, China University of Chinese Academy of Sciences Beijing China; 3 College of Biological Sciences and Agriculture, Qiannan Normal College for Nationalities, Duyun 558000, China College of Biological Sciences and Agriculture, Qiannan Normal College for Nationalities Duyun China; 4 Agriculture and Forestry Institute of Southwestern Guizhou, Xingyi 100049, China Agriculture and Forestry Institute of Southwestern Guizhou Xingyi China

**Keywords:** Molecular phylogeny, morphology, plastid CDS, species diversity, taxonomy

## Abstract

To clarify the taxonomic identities of newly-collected *Selaginella* specimens from Guizhou, south-western China, we briefly reviewed the *Selaginella* species recorded in Guizhou and conducted a study integrating morphological comparisons and phylogenetic analyses. The specimens from a total of 26 *Selaginella* populations which we collected in 2024 were grouped into 16 taxonomic entities, based on morphological comparisons, including ten species with established names and six taxa of uncertain identity. Phylogenetic analyses, based on plastid regions (*rbcL*, *atpI*, *psbA*) with broad sampling and plastid CDS with focused sampling, confidently placed 15 newly-collected specimens within the phylogeny: one specimen (*S. davidii*) within the *Kungiselaginella* clade and the remaining 14 distributed amongst three subclades of the *Hypopterygiopsis* clade. By integrating morphology and molecular evidence, we recognise one new species (*S. dushanensis*), one new provincial record (*S. parachrysocaulos*) to Guizhou and four cryptic species (*S. amblyphylla*, *S. qingchengshanensis* and two lineages of *S. vaginata*); where “cryptic species” denotes morphologically indistinguishable, but phylogenetically distinct lineages. Amongst these, *S. amblyphylla* and *S. qingchengshanensis* are also new records to Guizhou. The new species *S. dushanensis* is described and illustrated. It is morphologically very similar to *S. monospora* and *S. submonospora*, but differs mainly in median leaves with a long-aristate apex. As a result, the known diversity of *Selaginella* in Guizhou now records 43 species, including the four cryptic and one putative new taxon (*S. cf. xipholepis*). Further studies are needed to confirm the identities of the cryptic species and the remaining unidentified taxon.

## Introduction

*Selaginella* P.Beauv. (Selaginellaceae), the largest genus of extant lycophytes, comprises approximately 760 species across all continents of the world, except Antarctica (Hassler 1994–2025), with most species distributed in tropical and subtropical regions (Jermy 1990). The genus represents a monophyletic group characterised by the stele lying in an air-filled cavity and the megasporangia producing only four megaspores (Jermy 1990; Kenrick and Crane 1997). Species of *Selaginella*, predominantly medium-sized, inhabit ecologically diverse habitats (including arid and alpine zones) (Weststrand and Korall 2016a) and exhibit diverse growth appearances: erect, prostrate, climbing or rosette-forming (Zhou and Zhang 2015; Yang et al. 2023). Based on morphology and distribution, *Selaginella* has traditionally been subdivided into five subgenera (Jermy 1986; Jermy 1990). Molecular phylogenetic analyses, however, revealed that only two minor subgenera — Subg. *Selaginella* (two species) and Subg. *Tetragonostachys* (ca. 50 species) are monophyletic, while the majority of proposed subgenera lack molecular support for monophyly (Weststrand and Korall 2016b). On the base of phylogenetic analysis of plastid sequences, seven subgenera (Weststrand and Korall 2016b) or up to 20 genera were proposed within the traditional *Selaginella* (Zhou and Zhang 2023; Zhou et al. 2025).

Guizhou, a mountainous province of 176,200 km^2^ land area in south-western China, harbours rich species of *Selaginella*. This is attributed to its subtropical monsoon climate, karst-dominated topography and heterogeneous microhabitats. The species of *Selaginella*, as well as other lycophytes and ferns, have been relatively well studied compared to those in other provinces of China. As early as the 1910s, Léveillé (1915) recorded 16 species of *Selaginella* in Guizhou. By 2001, Wang and Wang (2001) had recognised 34 species of *Selaginella*, based on their lifelong research focusing on the biodiversity of lycophytes and ferns within Guizhou. Recent revisions by Wang and Pan (2018) increased this number to 41, including 40 native species and one introduced African species, *S. kraussiana* A.Braun. Amongst the 40 native species to Guizhou, *S. leptophylla* Baker should be treated as a synonym of *S. aristata* Spring (Chang et al. 2012). The three species, *S. gebaueriana* Hand.-Mazz., *S. xishuiensis* G.Q.Gou & P.S.Wang and *S. compta* Hand.-Mazz., are taxonomically disputed. *Selaginella gebaueriana* was regarded as conspecific with *S. davidii* Franch., while *S. xishuiensis* and *S. compta* were synonymised within *S. vaginata* Spring by Zhang et al. (2013). In addition, several taxonomic corrections in recent years have been made to *Selaginella* species which were recorded by Wang and Pan (2018) in Guizhou. For instance, *S. sanguinolenta* (L.) Spring is confirmed to occur from the Russian Far East to northern China; specimens previously recorded as *S. sanguinolenta* in Guizhou (Wang and Wang 2001; Wang and Pan 2018) are referable to *S. jacquemontii* Spring (Zhang et al. 2023). *Selaginella shensiensis* Christ, previously synonymised with *S. nipponica* Franch. & Sav. (Zhang 2004; Zhang et al. 2013), is resurrected as an independent species, based on molecular evidence; and *S. longistrobilina* P.S.Wang, X.Y.Wang & Li Bing Zhang described from Guizhou (Zhang et al. 2012) is subsumed under *S. shensiensis* (Zhang et al. 2021). Similarly, *S. hezhangensis* P.S.Wang & X.Y.Wang, which was described from Guizhou (Wang and Wang 1996) and subsequently synonymised with *S. heterostachys* Baker (Zhang 2004; Zhang et al. 2013), is resurrected, based on phylogenetic analysis of plastid sequences (Shalimov and Zhang 2022). Additionally, both *S. sichuanica* H.S.Kung and *S. daozhenensis* Li Bing Zhang, Q.W.Sun & Jun H.Zhao are synonymised under *S. labordei* Hieron. ex Christ by Zhang and Zhang (2022). Therefore, the number of *Selaginella* species currently recognised as native to Guizhou is revised to 37, excluding the alien *S. kraussiana* and the three disputed species (*S. gebaueriana*, *S. xishuiensis* and *S. compta*) (Table 1). This revised count represents approximately 37% of the total *Selaginella* species documented in China. Amongst the 37 species, eleven are currently endemic to China, including *S. spinulosovena* G.Q.Gou & P.S.Wang and *S. wangpeishanii* Li Bing Zhang, H.He & Q.W.Sun, which are poorly documented as they are exclusively represented by type specimens to date. Under the seven-subgenus classification of Weststrand and Korall (2016b), all Guizhou *Selaginella* species belong to Subg. *Stachygynandrum*, with the exception of *S. remotifolia* Spring which is in Subg. *Gymnogynum*. By contrast, under the classification of 20 genera (more precisely, clades) proposed by Zhou and Zhang (2023), nearly half of the Guizhou species fall within the *Hypopterygiopsis* clade, seven species in the *Didiclis* clade, four in the *Kungiselaginella* clade and one or two in each of other five clades (Table 1).

**Table 1.** Checklist of native *Selaginella* species to Guizhou, China and their infrageneric assignments.

Species	Geographical distribution	Subgenus (Weststrand and Korall 2016b)	Clade (Zhou and Zhang 2023)
*Selaginella albociliata* P.S.Wang	China (GX, GZ)	* Stachygynandrum *	*Hypopterygiopsis*
*S. aristata* Spring	SW China to N Australia	Stachygynandrum	*Hypopterygiopsis*
*S. bodinieri* Hieron.	China (SW to central)	Stachygynandrum	*Hypopterygiopsis*
*S. chaetoloma* Alston	China (GX, GZ)	Stachygynandrum	*Hypopterygiopsis*
*S. chrysocaulos* (Hook. & Grev.) Spring	E Himalaya to W Malaysia	Stachygynandrum	*Hypopterygiopsis*
*S. drepanophylla* Alston	China (GX, GZ, YN)	Stachygynandrum	*Hypopterygiopsis*
*S. effusa* Alston	SW China; Vietnam	Stachygynandrum	*Hypopterygiopsis*
*S. heterostachys* Baker	E Asia to Vietnam	Stachygynandrum	*Hypopterygiopsis*
*S. hezhangensis* P.S.Wang & X.Y.Wang	China (GZ, SC, YN)	Stachygynandrum	*Hypopterygiopsis*
*S. kouytcheensis* H.Lév.	China (GZ, YN)	Stachygynandrum	*Hypopterygiopsis*
*S. labordei* Hieron. ex Christ	China, Myanmar	Stachygynandrum	*Hypopterygiopsis*
*S. megaphylla* Baker	SW China to E Himalaya	Stachygynandrum	*Hypopterygiopsis*
*S. monospora* Spring	S China to E Himalaya	Stachygynandrum	*Hypopterygiopsis*
*S. repanda* (Desv.) Spring	E Himalaya to Malesia	Stachygynandrum	*Hypopterygiopsis*
*S. spinulosovena* G.Q.Gou & P.S.Wang	China (GZ)	Stachygynandrum	*Hypopterygiopsis*
*S. vaginata* Spring	E Himalaya to Indochina	Stachygynandrum	*Hypopterygiopsis*
*S. wangpeishanii* L.B.Zhang, H.He & Q.W.Sun	China (GZ)	Stachygynandrum	*Hypopterygiopsis*
*S. xipholepis* Baker	China (GD, GX, GZ, JX)	Stachygynandrum	*Hypopterygiopsis*
*S. braunii* Baker	China, Myanmar	Stachygynandrum	*Didiclis*
*S. delicatula* (Desv.) Alston	E Himalaya to New Guinea	Stachygynandrum	*Didiclis*
*S. helferi* Warb.	E Himalaya to Indochina	Stachygynandrum	*Didiclis*
*S. mairei* H.Lév.	SW China to Sumatra	Stachygynandrum	*Didiclis*
*S. picta* A.Braun ex Baker	E Himalaya to Indochina	Stachygynandrum	*Didiclis*
*S. uncinata* (Desv.) Spring	China, Japan, Vietnam	Stachygynandrum	*Didiclis*
*S. willdenowii* (Desv.) Baker	SW China to N Australia	Stachygynandrum	*Didiclis*
*S. biformis* A.Braun ex Kuhn	E Himalaya to New Guinea	Stachygynandrum	*Kungiselaginella*
*S. davidii* Franch.	China (SW to central)	Stachygynandrum	*Kungiselaginella*
*S. involvens* (Sw.) Spring	Tropical & subtropical Asia	Stachygynandrum	*Kungiselaginella*
*S. moellendorffii* Hieron.	E Asia to Vietnam	Stachygynandrum	*Kungiselaginella*
*S. doederleinii* Hieron.	E Asia, Indochina, Borneo	Stachygynandrum	*Chuselaginella*
*S. trachyphylla* Hieron.	S China to Indochina	Stachygynandrum	*Chuselaginella*
*S. nipponica* Franch. & Sav.	E Asia	Stachygynandrum	*Lycopodioides*
*S. shensiensis* Christ	China (GZ to BJ & HB)	Stachygynandrum	*Lycopodioides*
*S. jacquemontii* Spring	SW China to W Himalaya	Stachygynandrum	*Boreoselaginella*
*S. pulvinata* (Hook. & Grev.) Maxim.	E Himalaya to Indochina	Stachygynandrum	*Pulviniella*
*S. tamariscina* (P.Beauv.) Spring	E Asia to Philippines	Stachygynandrum	*Pulviniella*
*S. remotifolia* Spring	E Himalaya to New Guinea	* Gymnogynum *	*Gymnogynum*

To characterise morphological variation within *Selaginella* species distributed across Guizhou and adjacent southern China (Guangxi, Guangdong), we performed field observations in central-to-southern Guizhou in 2024, focusing on intraspecific polymorphism in key diagnostic traits. During field surveys, we documented 26 populations representing about 16 species of *Selaginella*. Amongst these, several species widespread in south-western China were easily identified, including *S. aristata*, *S. bodinieri* Hieron., *S. davidii*, *S. effusa* Alston, *S. heterostachys*, *S. involvens* (Sw.) Spring, *S. moellendorffii* Hieron., *S. monospora* Spring and *S. remotifolia*. However, several populations presented challenges in species identification. To clarify the identities of these populations, we conducted comprehensive morphological comparisons and phylogenetic analyses of plastid sequences. The findings are reported herein.

## Materials and methods

### Morphological study

Morphological observations were based on living plants in the wild, plants transplanted in greenhouse from the wild and herbarium specimens. All species of *Selaginella* recorded in China and adjacent regions were considered in morphological comparisons. The *Selaginella* specimens deposited in Herbaria BCU, BKF, IBSC and HNU were all carefully examined by us. In addition, we examined digitised specimens, especially type specimens, of all species morphologically similar to unidentified Guizhou specimens by accessing online databases of Herbaria B, BM, BR, CDBI, E, GH, IBSC, K, KUN, LD, MO, NY, P, PE, S, TAIF, UC and WU. Herbarium abbreviations follow Thiers (2020). For the study of both herbarium specimens and living plants, we focused on the states and the variations of plant habit, stem (size of diameter, colour, branching pattern), leaves on all parts (size, shape, arrangement), pattern of strobilus and ornamentations of megaspores and microspores, which are characters frequently used by previous authors (Wang and Wang 2001; Chang et al. 2012; Zhang et al. 2013).

For spore morphology observation, mature spores were directly mounted on aluminium stubs using conductive carbon tape. Samples were sputter-coated with a 10 nm platinum layer (SuPro Instruments Mini Coater) and imaged with a JEOL JSM-IT210 scanning electron microscope (SEM) at 15 kV accelerating voltage. All procedures were performed at the Public Laboratory of South China Botanical Garden, Chinese Academy of Sciences. Voucher specimens were deposited in Herbarium IBSC.

### Taxon sampling, sequencing and plastome assembly

To provide clues on the identifications of the unidentified specimens from Guizhou, we conducted phylogenetic analyses of *Selaginella*, based on three plastid regions (*rbcL*, *atpI*, *psbA*). We sampled 82 accessions of about 40 species covering the unidentified Guizhou specimens, their putative relatives and representatives of several major clades, identified by Zhou and Zhang (2023). Of the 82 accessions, eighteen were newly sequenced including 15 accessions newly collected from Guizhou, two accessions (each one of *S. bisulcata* Spring and *S. vaginata*) from adjacent provinces of China and one accession of *S. amblyphylla* from Thailand. The 15 newly-sequenced accessions from Guizhou include seven accessions of five identified species (*S. amblyphylla* Alston, *S. aristata*, *S. davidii*, *S. heterostachys*, *S. vaginata*) and eight unidentified accessions which are provisionally labelled *S.* sp.1 to *S.* sp.6 (Table 2).

**Table 2.** Species determination of *Selaginella* specimens newly collected in Guizhou, China.

Specimen (coll. no)	Location	Determination based on morphology	Position in plastid-based tree	Determination based on morphology and molecular analysis*
D6612	Duyun	*S. davidii*	*Kungiselaginella*	/
D6630	Xingyi	*S. davidii*	*Kungi*.	/
D6633	Xingyi	*S. davidii*	*Kungi*.	/
D6635	Xingyi	*S. davidii*	*Kungi*.	/
D6656	Xingyi	*S. davidii*	*Kungi*.	davidii
D6626	Anshun	*S. involvens*	*Kungi*.	/
D6625	Anshun	*S. moellendorffii*	*Kungi*.	/
D6736	Anshun	*S. remotifolia*	*Gymnogynum*	/
D6740	Dushan	*S. monospora*	*Hypopterygiopsis*-2	/
D6614	Duyun	*S. aristata*	*Hypo*.-2	*S. aristata*
D6652	Anlong	*S. aristata*	*Hypo*.-2	*S. aristata*
D6632	Xingyi	*S. amblyphylla*	*Hypo*.-3	*S. amblyphylla* (cryptic)
D6610	Duyun	*S. bodinieri*	*Hypo*.-3	/
D6616	Duyun	*S. bodinieri*	*Hypo*.-3	/
D6648	Anlong	*S. bodinieri*	*Hypo*.-3	/
D6659	Xingyi	*S. heterostachys*	*Hypo*.-3	*S. heterostachys*
D6638	Xingyi	*S. vaginata*	*Hypo*.-1	*S. vaginata* (cryptic)
D6662	Xingyi	*S. vaginata*	*Hypo*.-1	*S. vaginata* (cryptic)
D6631	Xingyi	sp.1, cf. labordei	*Hypo*.-1	*S. cf. xipholepis*
D6675	Xingyi	sp.1, cf. labordei	*Hypo*.-1	*S. cf. xipholepis*
D6754	Dushan	sp.2, cf. vaginata	*Hypo*.-1	*S. vaginata* (cryptic)
D6661	Xingyi	sp.3, cf. effusa	*Hypo*.-2	*S. qingchengshanensis* (cryptic)
D6664	Xingyi	sp.3, cf. effusa	*Hypo*.-2	*S. qingchengshanensis* (cryptic)
D6749	Dushan	sp.4, cf. monospora	*Hypo*.-2	*S. effusa*
D6750	Dushan	sp.5, cf. monospora	*Hypo*.-2	*S. dushanensis* (sp. nov.)
D6641	Xingyi	sp.6, cf. aristata	*Hypo*.-3	*S. parachrysocaulos* (new record)

*The symbol “/” is used for samples not included in the molecular analysis.

Total genomic DNA was extracted from silica-dried leaf tissues using the Magnetic Plant Genomic DNA Kit (Tiangen Biotech, Beijing, China). High-quality DNA samples were outsourced for library preparation and 150-bp paired-end sequencing on the MGI DNBSEQ-T7 platform (Annoroad Gene Technology, Beijing). Raw reads were assembled *de novo* with GetOrganelle v.1.7.7.0 (Jin et al. 2020), using reference plastomes of *S. nipponica* (MK293725 and MK293726) and *S. uncinata* (Desv.) Spring (MG272483) (Zhang and Zhang 2022). Although the complete plastid genomes could not be assembled for all samples, target plastid regions were still successfully extracted. The assembled plastomes were circularised and annotated in GENEIOUS v.9.0.2 (Kearse et al. 2012). From these, the plastid regions *rbcL*, *atpI* and *psbA* were extracted. Each individual plastid region was aligned using MAFFT v.7 (Katoh and Standley 2013) and the resulting alignments were trimmed with using trimAl v.1.4 (Capella-Gutierrez et al. 2009) to remove gap-rich sites. The individually aligned sequences for each accession were concatenated using PhyloSuite v.1.2.2 (Zhang et al. 2020), resulting in a final matrix of concatenated *rbcL*, *atpI* and *psbA* regions for 82 accessions. Voucher information and GenBank accession numbers of these plastid regions are listed in Suppl. material 1.

Previous phylogenetic studies of the *S. labordei* group, including our preliminary work and Zhang and Zhang (2022), have indicated that analyses, based on a few plastid regions, yield low resolution. To address this limitation and to clarify the phylogenetic position of sample *D6641*, we reconstructed a phylogeny using a matrix of 37 plastid coding DNA sequences (CDS) representing 32 species (including *D6641*). The procedures for CDS extraction, alignment and trimming followed the same protocols as described above. Details of these CDS and their specimen vouchers are provided in Suppl. material 2, while the aligned plastid CDS matrix is available in Suppl. material 3.

### Phylogenetic analyses

The two matrices, one of concatenated *rbcL*, *atpI* and *psbA* sequences from 82 accessions and the other of 37 plastid CDS, were subjected to phylogenetic analysis using Maximum Likelihood (ML), Maximum Parsimony (MP) and Bayesian Inferences (BI). The MP analysis was performed in PAUP* ver. 4.0a169 (Swofford 1991) under the following conditions: all characters weighted equally and gaps treated as missing data. Heuristic searches with 1000 random addition sequence replicates were performed, each employing tree bisection-reconnection (TBR) branch swapping and saving a maximum of 100 trees per replicate. MP bootstrap support (MPBS) was evaluated, based on 1000 replicate analyses. The best-fitting substitution models for the ML and BI analyses were selected using software tool “Smart Model Selection” (SMS) (Lefort et al. 2017). ML tree searches and bootstrap analysis (1000 replicates) under the GTRGAMMA model were performed with RAxML-HPC2 on the CIPRES Science Gateway (Miller et al. 2010; Stamatakis 2014). Bayesian Inference (BI) was carried out using MrBayes 3.2.6 (Ronquist et al. 2012). The analysis ran for 10 million generations, sampling one tree every 1000 generations from four simultaneous Markov Chain Monte Carlo (MCMC) chains. After discarding the first 25% of samples as burn-in, the remaining trees were used to calculate posterior probabilities (PP).

## Results

### Morphology Comparison

Our morphological identification of the 26 Guizhou populations revealed 16 taxonomic entities. These included multiple populations of *S. aristata* (2), *S. bodinieri* (3), *S. davidii* (5) and *S. vaginata* (2). In addition, six species (*S. amblyphylla*, *S. heterostachys*, *S. involvens*, *S. moellendorffii*, *S. monospora* and *S. remotifolia*) were each represented by a single population collected during our recent surveys. The remaining eight populations, representing six taxonomic entities (*S.* sp.1 to *S.* sp.6), could not be identified to species level, based on morphological characters (Table 2).

### Phylogenetic Analyses

The concatenated alignment of three plastid regions (*rbcL*, *atpI*, *psbA*) for 82 accessions yielded a matrix of 3,241 sites, comprising 2,401 constant and 588 parsimony-informative characters. The Maximum Likelihood (ML) tree had a likelihood score of -12552.37. The most parsimonious (MP) tree had a length of 1,541 steps (CI = 0.606; RI = 0.808; RC = 0.489).

The 37 plastid CDS matrix contained 27,519 sites, with 11,427 constant and 13,957 parsimony-informative characters. Its MP tree was 45,603 steps long (CI = 0.481; RI = 0.821; RC = 0.395). The ML tree had a likelihood score of -238615.52.

**Figure 1.**
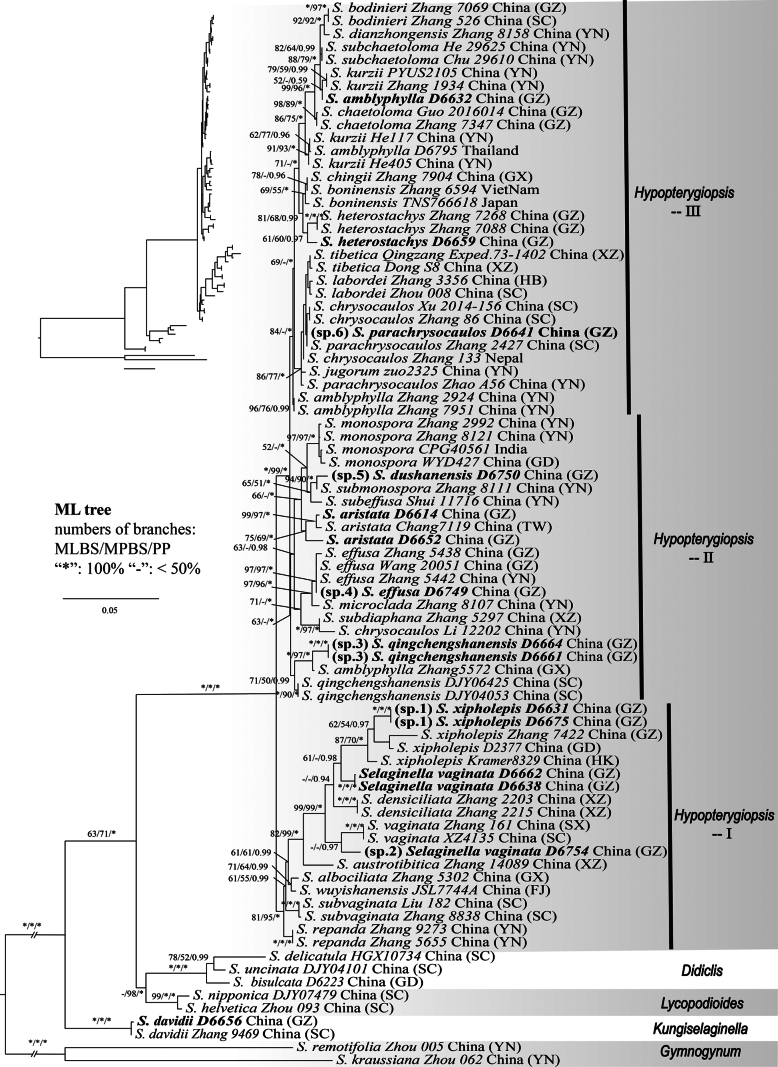
Maximum Likelihood phylogeny of *Selaginella*, based on concatenated sequences of the plastid *rbcL*, *atpI* and *psbA*. Five major clades, which were recognised as genera by Zhou and Zhang (2023), are indicated on the right. Accessions from Guizhou newly sequenced in this study are highlighted in bold.

Phylogenetic analyses of the three plastid regions using ML, MP and BI methods yielded generally congruent topologies, resolving all accessions into five major clades. Most accessions fell within the well-supported monophyletic clade *Hypopterygiopsis*, which was further subdivided into three subclades. Within *Hypopterygiopsis*, Clade I (accessions from *S. repanda* to *S. xipholepis*; Fig. 1) was strongly supported as sister to a clade comprising Clade II and III. Clade II (from *S. qingchengshanensis* to *S. monospora*) and III (from *S. amblyphylla* to *S. bodinieri*) were themselves strongly supported as monophyletic and sister to each other under BI (Fig. 1).

Phylogenetic placement revealed that, amongst the 15 newly-sequenced accessions from Guizhou, only *D6656* (identified as *S. davidii*) was resolved within the *Kungiselaginella* clade. The remaining 14 accessions were all placed within the *Hypopterygiopsis* clade, distributed across its subclades as follows: five in Clade I, six in Clade II and three in Clade III.

Within Clade I of *Hypopterygiopsis*, the five newly-sequenced accessions represent three species: *S. vaginata* (*D6638*, *D6662*), *S.* sp. 1 (*D6631*, *D6675*) and *S.* sp. 2 (*D6754*). *Selaginella* sp. 2 was resolved as sister to two accessions of *S. vaginata* from western to central China (Sichuan, Shaanxi). The two Guizhou accessions of *S. vaginata* formed a clade that was sister to a lineage comprising *S. xipholepis* and *S.* sp. 1. The two accessions of *S.* sp. 1 formed a strongly supported monophyletic group (MLBS = 100%, MPBS = 100%, PP = 1.0), which was sister to *S. xipholepis*.

Within Clade II of *Hypopterygiopsis*, the six newly-sequenced Guizhou accessions represent four species: *S. aristata* (*D6614* & *D6652*), *S.* sp. 3 (*D6661* & *D6664*), *S.* sp. 4 (*D6749*) and *S.* sp. 5 (*D6750*). The two Guizhou accessions of *S. aristata* formed a clade with one accession from Taiwan (*Chang 7119*). *Selaginella* sp. 3 was strongly supported as sister to an accession of *S. amblyphylla* (*Zhang 5572*) from Guangxi, China (MLBS = 100%, MPBS = 97%, PP = 1.0). *Selaginella* sp. 4 was resolved as sister to *S. effusa* (MLBS/MPBS = 97%, PP = 1.0) and *S.* sp. 5 was strongly supported as sister to *S. submonospora* Shalimov & X.C.Zhang (MLBS = 94%, MPBS = 90%, PP = 1.0).

Within Clade III of *Hypopterygiopsis*, the three newly-sequenced accessions from Guizhou represent these three species: *S. amblyphylla* (*D6632*), *S. heterostachys* (*D6659*) and *S.* sp. 6 (*D6641*). Accession *D6632* was resolved as sister to several accessions of *S. kurzii* Baker from Yunnan, albeit with weak support (MLBS = 52%, MPBS < 50%, PP = 0.59). Accession *D6659* clustered with two other Guizhou accessions of *S. heterostachys* Baker (*Zhang 7088*, *7268*). *Selaginella* sp. 6 (*D6641*) was placed in a clade containing *S. chrysocaulos* (Hook. & Grev.) Spring, *S. parachrysocaulos* M.H.Zhang & X.C.Zhang, *S. labordei*, *S. tibetica* Ching & S.K.Wu and *S. jugorum* Hand.-Mazz. However, the phylogenetic relationships within this clade, including the position of *S.* sp. 6, remained unresolved in the analysis, based on the three concatenated plastid regions (*rbcL*, *atpI*, *psbA*).

**Figure 2.**
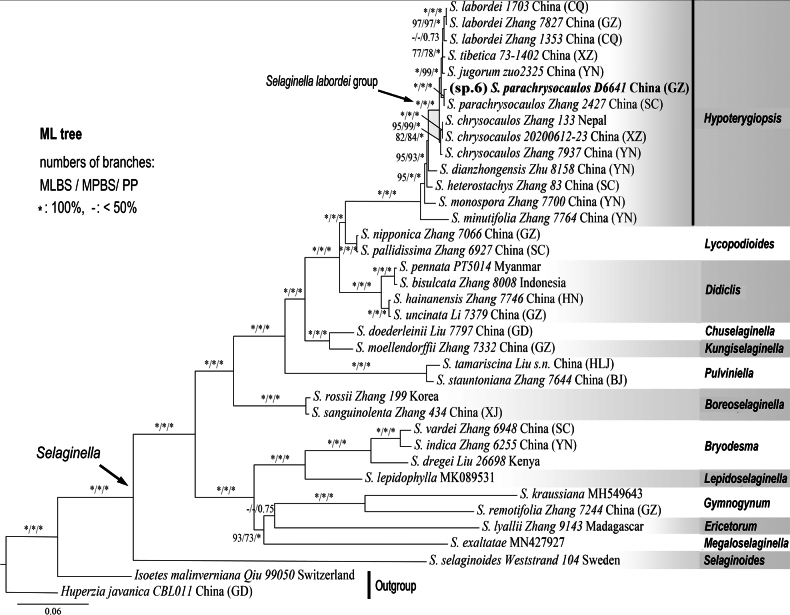
Maximum Likelihood phylogeny of *Selaginella* with emphasis on *S. labordei* group, based on plastid coding DNA sequences (CDS). The position of *S. parachrysocaulos* (accession *D6641*) is highlighted in bold. The clades, recognised as genera by Zhou and Zhang (2023), are labelled on the right.

To resolve this ambiguity, we analysed a small dataset of 37 plastid CDS, which yielded a fully resolved topology for the critical clade (Fig. 2). Within this robust framework, the unidentified *S.* sp. 6 formed a strongly supported clade (MLBS/MPBS = 100%, PP = 1.0) with an accession of *S. parachrysocaulos* (*Zhang 2427*). This well-supported lineage was recovered as sister to a clade containing *S. labordei*, *S. jugorum* and *S. tibetica*.

## Discussion

### Morphological identification of *Selaginella* species supported by molecular analyses

In this study, we identified the 26 populations collected in the field of Guizhou as 16 taxonomic entities. Amongst these, fifteen populations representing 11 distinct entities were sampled for molecular phylogenetic analysis. Molecular phylogenetic analyses resolved these populations into 11 lineages, a finding that is fully congruent with the results of morphological identification. In all cases where multiple populations identified as the same morphological entity, they formed monophyletic terminal clades. Specifically, the pairs *S. vaginata* (*D6638*, *D6662*), *S.* sp. 1 (*D6631*, *D6675*) and *S.* sp. 3 (*D6661*, *D6664*) each clustered together with strong support. A single exception was observed in *S. aristata*: specimens *D6614* and specimens *D6652*, despite morphologically congruent and part of a monophyletic *S. aristata* clade, were not direct sisters. This suggests the presence of genetic divergence amongst populations within this species. Compared to other samples of *S. aristata*, specimen *D6652* exhibited greater sequence divergence in plastid regions analysed. The strong concordance between molecular phylogeny and morphological circumscription validates the significant taxonomic value of several characters frequently employed in previous studies (Wang and Wang 2001; Chang et al. 2012; Zhang et al. 2013). These include plant habit (erect vs. prostrate), sporophyll dimorphism and the detailed morphology of leaves (median, lateral, axillary and sporophylls), such as their shape, margin, apex and base, as well as spore colour and ornamentations.

### Pervasive cryptic species in *Selaginella* revealed by molecular phylogenetics

Our integrated morphological and molecular analyses reveal that cryptic species are prevalent across *Selaginella*. Here, the concept of cryptic species refers to those species that display a consistent morphology (or lacking obvious difference in morphology), but exhibit interspecific genetic divergence in the phylogenetic analysis, leading accessions from different populations to represent distinct phylogenetic clades. As shown in the analysis of 82 accessions of around 40 species (Fig. 1), seven species (*S. amblyphylla*, *S. boninensis*, *S. chrysocaulos*, *S. kurzii*, *S. qingchengshanensis*, *S. vaginata* and *S. xipholepis*) were found to be non-monophyletic. For example, the newly-sequenced Guizhou accession of *S. amblyphylla* (*D6632*), accessions of *S. qingchengshanensis* (*D6661*, *D6664*) and those of *S. vaginata* (*D6662*, *D6638*, *D6754*) each potentially represents one or two cryptic species. We noted that the occurrence of cryptic species in *Selaginella* has been also frequently reported in previous studies. For example, a comprehensive analysis of *S. delicatula* across its geographical range resolved the samples into two distinct clades (Zhang and Zhang 2021); *S. labordei* was mentioned to be an aggregation (Hassler 1994–2025), encompassing three morphologically indistinguishable cryptic species (Zhang and Zhang 2022); in the study by Huang et al. (2022), six accessions of *S. effusa* were assigned to two separate clades; the accessions of *S. vaginata* analysed by Zhang et al. (2024) were resolved in three distinct clades.

### Withholding species establishment for cryptic lineages lacking diagnostic morphological characters

We contend withholding the establishment of new species for genetically distinct, but morphologically cryptic lineages in *Selaginella*. For instance, our analysis indicates that Guizhou accessions *D6638* and *D6662* represent a cryptic lineage within *S. vaginata*, while *D6661* and *D6664* represent a cryptic lineage within *S. qingchengshanensis* (Fig. 1). However, no consistent morphological or ecological differences have been observed to distinguish these lineages from their corresponding type specimens, nor are their distribution limits fully resolved. Establishing new species, based solely on molecular divergence, risks nomenclatural instability and complicates practical taxonomic communication. Therefore, we advocate for restraint and provisionally retain these accessions under established names until diagnostic morphological characters are identified.

It is also important to note that the putative cryptic lineages identified here are inferred exclusively from plastid data. The plastid sequence divergence underlying these lineages could stem from processes, such as hybridisation, incomplete lineage sorting (ILS) or reticulate evolution, which means that the plastid-based phylogeny may not accurately represent species relationships (Degnan and Rosenberg 2009; Gonçalves et al. 2019; Zhao et al. 2024). Therefore, it is necessary to conduct analyses of nuclear genome, particularly single-copy nuclear genes, for *Selaginella*. Such data will provide a robust framework for evaluating species relationships, which, when integrated with morphological, geographic and ecological evidence, will lead to more convincing taxonomic conclusions.

### Taxonomic identities of the taxa newly discovered in Guizhou

Our morphological comparison and phylogenetic analyses confirm that specimen *D6749* (provisionally labelled *S.* sp. 4) represents *S. effusa*, although it is considerably larger in individual size than typical *S. effusa* populations found in southern China. This finding suggests that specimens previously identified as *S. effusa* and clustered with samples of *S. monospora* are probably representatives of *S. monospora*, as exemplified by Gaoligong Shan Biodiversity Survey 22153 (GH) from north-western Yunnan (Weststrand and Korall 2016a) and *D5485* (IBSC) from south-eastern Yunnan (Huang et al. 2022). To our knowledge, *S. effusa* is indistinguishable from *S. monospora*, as some diagnostic characters (e.g. plant habit and size, sporophyll dimorphism) used to separate them (Wang and Wang 2001; Zhang 2004; Zhang et al. 2013) exhibit significant plasticity. However, the bearing pattern of lateral leaves on stems or branches, i.e. the extent of the acroscopic base of lateral leaf overlapping stem/branch, appears to be a useful character for separating *S. effusa* from *S. monospora*.

**Figure 3.**
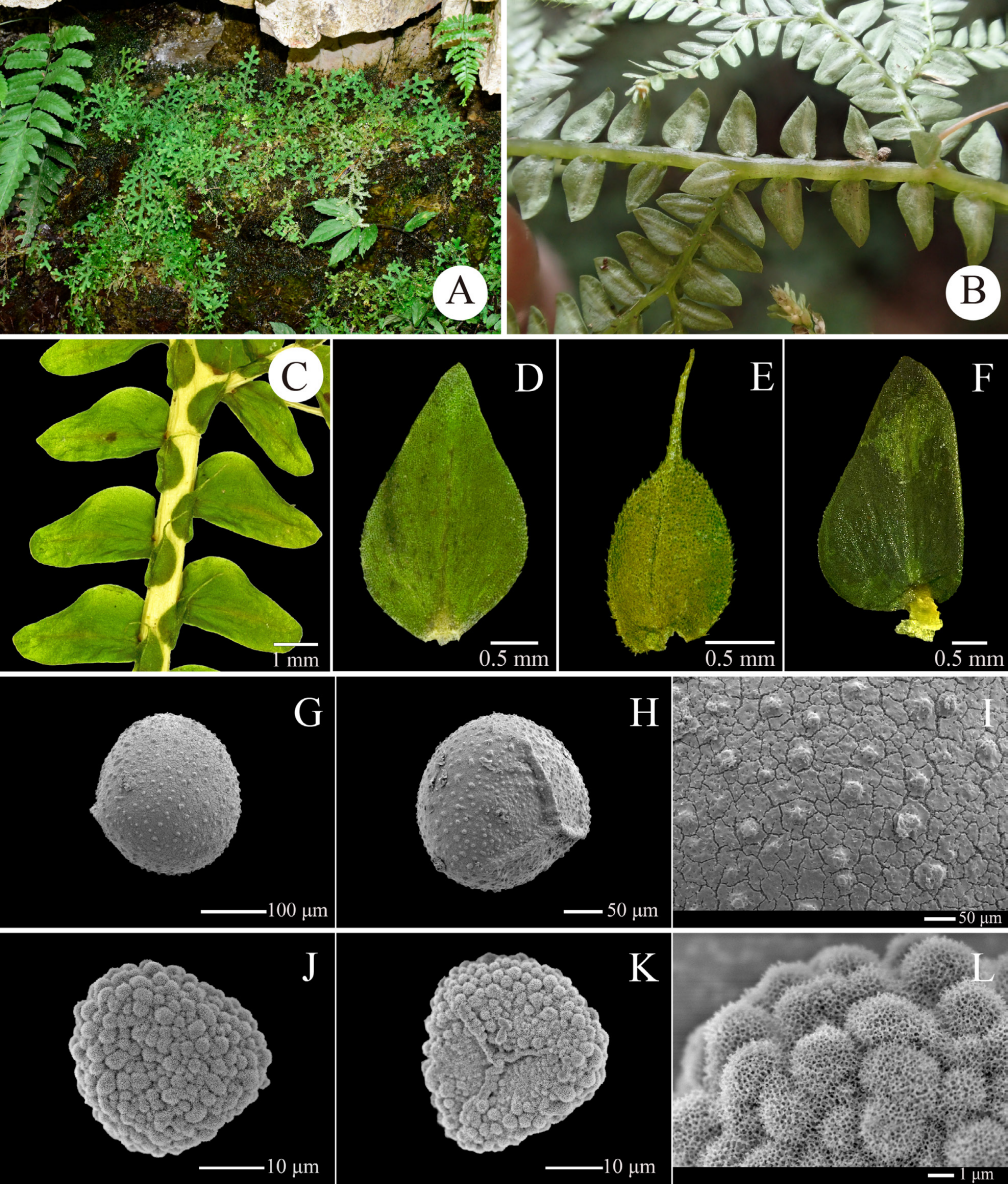
Morphology of *Selaginella dushanensis*. **A**. Habit; **B**. Portion of main stem with branches (lower view); **C**. Portion of main stem (upper view); **D**. Axillary leaf (upper view); **E**. Median leaf (upper view); **F**. Lateral leaf (upper view); **G**. Distal surface of megaspore; **H**. Proximal surface of megaspore; **I**. Detail of megaspore on distal surface; **J**. Distal surface of microspore; **K**. Proximal surface of microspore; **L**. Detail of microspore on distal surface (all from *D6750*).

*Selaginella* sp. 5 (voucher *D6750*) is here described as a new species, *S. dushanensis*, based on distinct morphological and phylogenetic evidence. *Selaginella dushanensis* is morphologically similar to *S. monospora* and *S. submonospora*. However, *S. dushanensis* differs from the latter two mainly by its median leaves with long aristate apices (Fig. 3E). This new species morphologically also resembles *S. effusa* and *S. subeffusa* Shalimov & X.C.Zhang, but differs distinctly in that the bases of its lateral leaves do not overlap the stems or branches on which they are borne (Fig. 3B). A morphological comparison of *S. dushanensis* with these similar species is provided in Table 3. Our phylogenetic analysis indicates that *S. dushanensis* is closely allied to *S. submonospora*; these two species form a sister relationship in the tree with high support values (Fig. 1). Apart from distinct differences in the shape of median leaf apex (acuminate vs. long-aristate) and the microsculpture on microspore, *S. submonospora* is currently known only from the East Himalaya (south-western Yunnan) (Shalimov and Zhang 2022), whereas *S. dushanensis* occurs in south-eastern Guizhou, China.

**Table 3.** Morphological comparison of *Selaginella dushanensis* with similar species.

	*S. dushanensis*	*S. effusa*	*S. subeffusa*	*S. monospora*	*S. submonospora*
Plant habit	Creeping; 10–30 cm long	Creeping or suberect; 10–45 cm long	Creeping or suberect; 15–30 cm long	Creeping; 30–50 cm long	Creeping; 10–20 cm long
Shape of axillary leaves	Elliptic to ovate	Ovate to ovate-triangular	Ovate to ovate-triangular	Ovate	Elliptic
Shape of median leaves	Ovate or broadly ovate	Ovate-elliptic	Ovate	Ovate	Broadly ovate
Median leaf apex	Long-aristate	Aristate	Aristate	Aristate or cuspidate	Acuminate
Acroscopic base of lateral leaves	Not overlapping stem	Strongly overlapping stem	Strongly overlapping stem	Slightly overlapping stem	Not overlapping stem
Megaspore colour	Pinkish to reddish-brown	White-yellow	White	White	Pinkish
Microspore ornamentation	Verrucate	Verrucate, spherulate or tuberculate	Verrucate	Verrucate or spherulate	Verrucate
Microspore microsculptures	Honeycomb-like	(lacking data)	Perforate	Fine reticulate	Vermiculate

Specimen *D6641* (provisionally labelled *S.* sp. 6) is supported as a representative of *S. parachrysocaulos* by our phylogenetic analysis and morphological comparison. *Selaginella parachrysocaulos* is morphologically similar to *S. chrysocaulos*, both possessing a rare character in *Selaginella*: the presence of tuber-like rhizomes (Zhang and Zhang 2022). These two species are morphologically almost indistinguishable, differing primarily in microspore surface ornamentation: honeycomb-like in *S. parachrysocaulos* versus smooth or spinulose in *S. chrysocaulos* (Zhang and Zhang 2022). *Selaginella parachrysocaulos* was previously known only from the border region between northern Yunnan and south-western Sichuan, China (Zhang and Zhang 2022); its occurrence in southern Guizhou (represented by *D6641*) represents a new distribution record.

The taxonomic identity remains unclear for the five species: *S.* sp. 1 (*D6631*, *D6675* as *S. xipholepis*), *S.* sp. 2 (*D6754* as *S. vaginata*), *S.* sp. 3 (*D6661*, *D6664* as *S. qingchengshanensis*), the species represented by *D6632* (as *S. amblyphylla*) and that by *D6638* and *D6662* (as *S. vaginata*).

Our phylogenetic analysis resolved the Guizhou accessions of *S.* sp. 1 as sister to those of *S. xipholepis* from southern China. However, the two are morphologically distinct. The Guizhou accessions (*S.* sp. 1) possess sterile leaves with margins toothed or ciliolate only at the base, whereas those of typical *S. xipholepis* are ciliate from the leaf base to at least the middle region. Since *S. xipholepis* was originally described, based on specimens from southern China (Baker 1885), the Guizhou accessions (*D6631*, *D6675*) may represent an undescribed species. Nevertheless, because the morphological variations within *S. xipholepis* remain insufficiently understood, we refrain from formally describing *S.* sp. 1 as new at this time. A definitive conclusion requires future comprehensive study incorporating broader sampling and detailed morphological analysis.

In the phylogenetic tree, *S.* sp. 2 (*D6754*), collected from Dushan, Guizhou, formed a sister clade to accessions of *S. vaginata* from Sichuan and Shaanxi (e.g. *Zhang 161*, *XZ4135*). The observed morphology and plastid DNA differences between them suggest that *S.* sp. 2 is a close relative rather than a conspecific entity. In addition, two accessions from Xingyi, Guizhou (*D6638*, *D6662*), while morphologically similar to *S. vaginata*, were phylogenetically closer to *S. xipholepis*. This indicates that *D6638* and *D6662* also likely do not represent true *S. vaginata*. Given the lack of further evidence, we tentatively treat both the Dushan (*D6754*) and Xingyi (*D6638*, *D6662*) populations as cryptic species within the *S. vaginata* complex.

*Selaginella* sp. 3 (*D6661*, *D6664*) is morphologically similar to *S. effusa*, which was previously recorded occurring in Guizhou (Wang and Pan 2018), but differs in having ovate (vs. ovate-triangular) axillary leaves, lateral leaves slightly (vs. obviously) overlapping the stems and pinkish (vs. white-yellow) megaspores. Phylogenetically, however, it forms a clade with an accession labelled *S. amblyphylla* (*Zhang 5572*) from Guangxi and this combined clade is sister to *S. qingchengshanensis* which is currently only known in Sichuan (He et al. 2021). When examining the type specimens of *S. qingchengshanensis*, we found that the morphology of *S.* sp. 3 complies exactly with that of *S. qingchengshanensis*. Therefore, we suggest to tentatively regard *S.* sp. 3 as a cryptic species of the latter.

Although the specimen *D6632* (Fig. 5) shows no significant morphological differences with Thai specimen *S. amblyphylla* (Fig. 6), molecular phylogenetic analyses suggest that it is not the true *S. amblyphylla*, because the typical form of *S. amblyphylla* was described, based on specimens collected from Thailand (Alston 1934). In the phylogenetic tree (Fig. 1), the Guizhou accession *D6632* and the Thai accession *D6795* were placed in distinct clades, indicating substantial sequence divergence between them. At present, we tentatively consider *D6632* to represent a cryptic species related to *S. amblyphylla*.

### Taxonomic treatment

#### Selaginella dushanensis

Taxon classificationPlantaeSelaginellalesSelaginellaceae

J.K.Huang & S.Y.Dong sp. nov.

579C709D-0D2A-5929-803C-063E89A03E98

urn:lsid:ipni.org:names:77379348-1

Figs 3, 4

##### Type.

China • Guizhou: Dushan County, Jia Feng Yan; 25°59'15"N, 107°40'15"E, elev. 770 m, in evergreen broadleaf forest, 7 November 2024, *S.Y.Dong & J.K.Huang D6750* (holotype, IBSC, barcode 1047559; isotypes: IBSC-1047560, PE).

**Figure 4.**
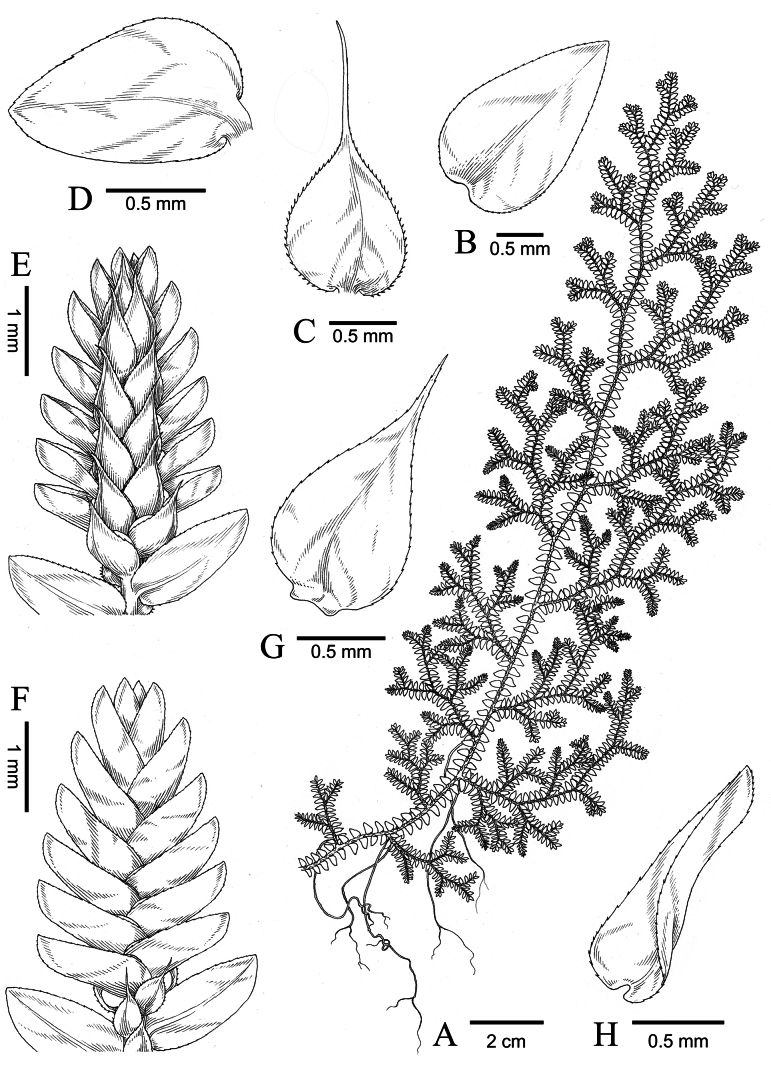
Illustrated morphology of *Selaginella dushanensis*. **A**. Habit; **B**. Axillary leaf; **C**. Median leaf **D**. Lateral leaf; **E**. Strobilus (lower view); **F**. Strobilus (upper view); **G**. Ventral sporophyll; **H**. Dorsal sporophyll (lower view) (drawn by Shuhan Li, based on *D6750*).

##### Diagnosis.

*Selaginella dushanensis* is similar to *S. monospora* and *S. submonospora*, but differs from the latter two mainly by its median leaves with long aristate apices (vs. apices acuminate in *S. submonospora* or short aristate in *S. monospora*). This new species is morphologically also similar to *S. effusa* and *S. subeffusa*, but differs by its lateral leaves with the upper bases not overlapping stems (vs. distinctly overlapping stems in the latter two).

##### Description.

Terrestrial, creeping, 10–30 cm long; ***rhizophores*** restricted throughout the creeping stems and branches, singly borne on ventral side in the axils of branches, reddish, glabrous; ***main stem*** branched from the base, terete, glabrous, stramineous in colour, 0.7–1.0 mm in diam., 5.0–8.0 mm wide including leaves; ***primary branches*** alternate, 1.0–2.5 cm apart along the main stem, once or twice pinnately branched, with ultimate branches including leaves being 3.0–5.0 mm wide; ***vegetative leaves*** herbaceous, dimorphic, arranged in four rows (two dorsal and two ventral); ***axillary leaves*** symmetrical, those on main stems slightly larger than those on branches, elliptic to ovate, 1.7–2.5 × 0.8–1.5 mm, base cuneate to obtuse, apex acuminate, margin denticulate; ***median leaves*** asymmetrical, slightly carinate, those on main stems larger than those on branches, slightly distant on main stems and imbricate on distal portions of stems and branches, ovate or broadly ovate, 0.8–1.7 × 0.6–1.3 mm, base obliquely shallowly cordate with the outer potion obviously narrower than the inner portion, margin denticulate or denticulate-ciliolate, apex long aristate (arista ca. 2/3 to as long as the leaf blade); ***lateral leaves*** asymmetrical, those on main stem larger than those on branches, distant on lower and middle portions, contiguous on distal portions of stems and branches, slightly deflected, ovate or ovate-oblong, 2.0–3.5 × 0.8–2.0 mm, margin denticulate, rounded at base, with acroscopic base enlarged, not or slightly overlapping stems and branches, apex acute; ***strobili*** solitary or pairs, terminal on the branches, compact, dorsiventrally complanate, 3.0–5.0 × 1.8–2.5 mm; sporophylls strongly dimorphic, resupinate (i.e. the smaller sporophylls in the ventral plane); ***dorsal sporophylls*** ovate-lanceolate, 1.1–1.8 × 0.3–0.5 mm base rounded, apex acute, margin denticulate, with the sporophyll-pteryx complete and denticulate; ***ventral sporophylls*** ovate-lanceolate, 0.8–1.2 × 0.3–0.6 mm, obviously carinate, base rounded, apex long acuminate, margin denticulate; ***megasporangia*** borne in basal portion of strobilus on lower side; ***megaspores*** pinkish to reddish-brown, subglobose, 220–280 μm in diam., surface papillate; ***microspores*** pale yellow, tetrahedral-globose, 24–32 μm in diam., surface verrucate with dense honeycomb-like microsculpture.

##### Distribution and habitat.

*Selaginella dushanensis* is currently known only from the type locality: Jia Feng Yan Forest in Dushan County, south-eastern Guizhou. The species occurs on wet rocks in montane evergreen broadleaved forest, along a perennial stream at elevations of 700–800 m.

##### Etymology.

The specific epithet dushanensis is derived Dushan County, south-eastern Guizhou, where the new species was first discovered.

##### Conservation status.

Currently, the only known population of *Selaginella dushanensis* is located in Dushan, Guizhou. The individuals are scattered along a stream bank for a distant of ca. 500 metres. We estimate that there are more populations of this species existing in south-eastern Guizhou and adjacent regions, which are to be discovered in the future. The exact population size and distribution range remain unclear. We provisionally assess its conservation status as Data Deficient (DD) according to IUCN Red List Categories and Criteria (IUCN 2024).

#### Selaginella amblyphylla

Taxon classificationPlantaeSelaginellalesSelaginellaceae

(cryptic species), a new record to Guizhou, China

A351AC69-2109-59A8-AEE0-67CAC34AE3E4

Fig. 5

Selaginella amblyphylla Alston, Bull. Fan Mem. Inst. Biol. Bot. 5: 287. 1934.

##### Type.

Thailand • Chiang Mai: Doi Angka, 2 December 1928, *H.M.Smith 357* (holotype, BM, barcode 000779901; isotypes: GH-00022032, US-00134348).

**Figure 5.**
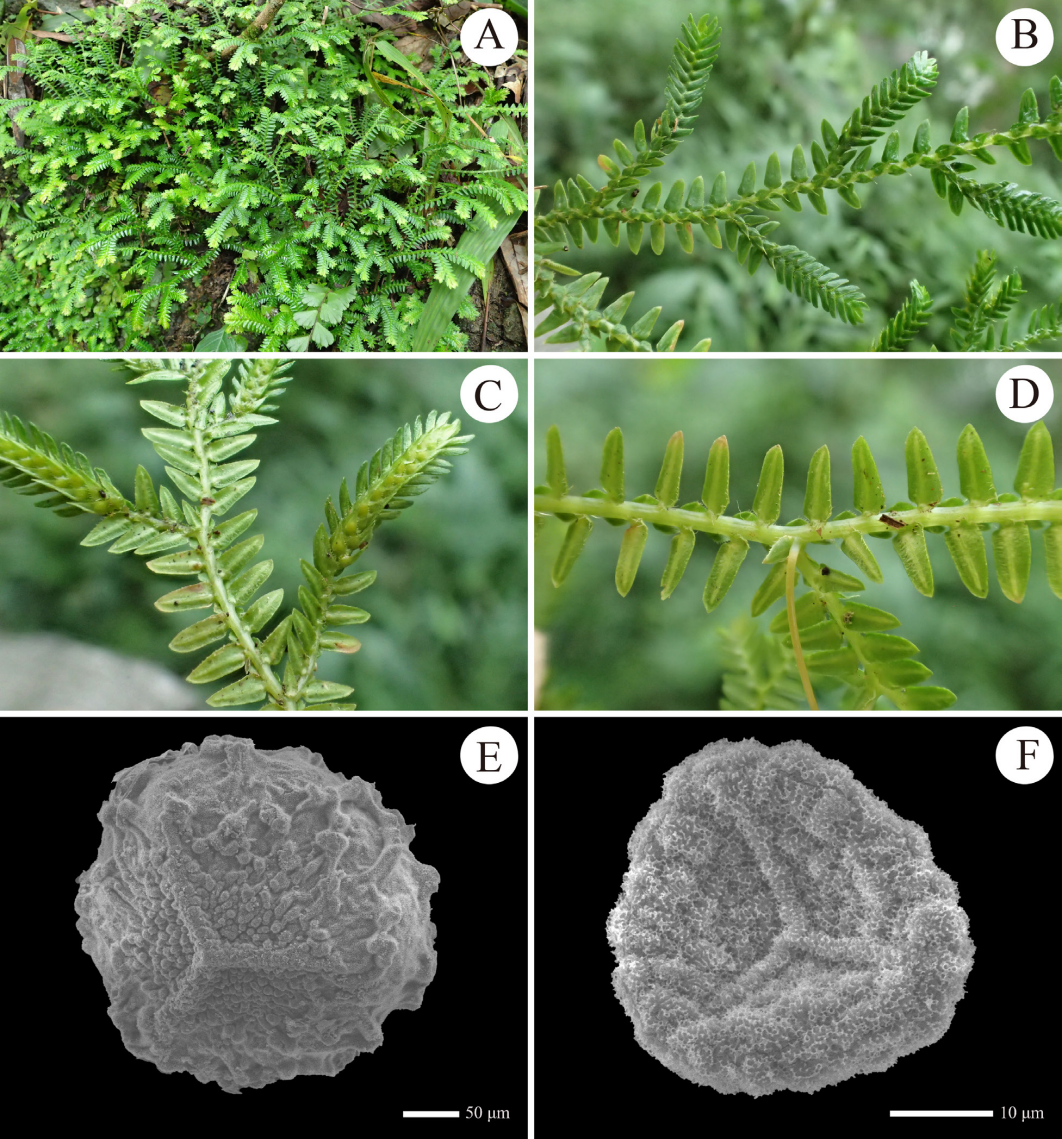
Morphology of *Selaginella amblyphylla* (cryptic species) from Guizhou, China. **A**. Habit; **B**, **C**. Distal stem with branches and strobili (B: upper view; C: lower view); **D**. Portion of main stem (lower view); **E**. Proximal surface of megaspore; **F**. Proximal surface of microspore (all from *D6632*).

**Figure 6.**
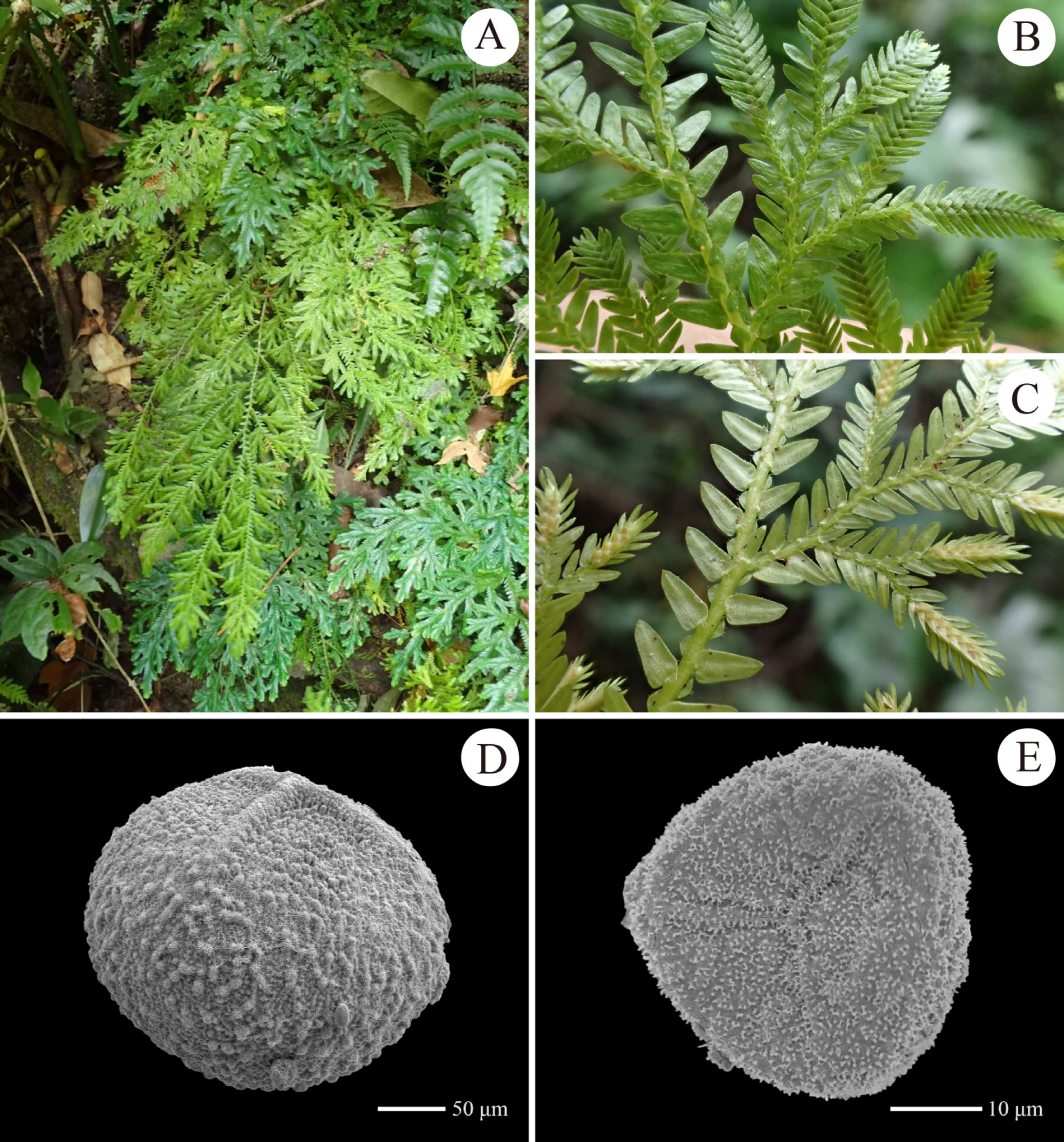
Morphology of *Selaginella amblyphylla* from Thailand. **A**. Habit; **B**, **C**. Distal stem with branches and strobili (**B**. Upper view; **C**. Lower view); **D**. Proximal surface of megaspore; **E**. Proximal surface of microspore (all from *D6795*).

##### Description.

Plant terrestrial, evergreen, creeping or suberect, 10–23 cm long; ***rhizophores*** at intervals throughout length of creeping stem and branches, borne on lateral side in axils of branches; ***main stem*** branched from near base, terete, glabrous, stramineous when dry, 0.3–0.6 mm in diam., 5.0–8.0 mm wide including leaves; ***primary branches*** alternate, 0.6–2.5 cm apart, once or twice pinnately branched, with ultimate branches including leaves 4.0–6.0 mm wide including leaves. ***vegetative leaves*** herbaceous, dimorphic, arranged in four rows; ***axillary leaves*** symmetrical, ovate-lanceolate, 1.5–2.5 × 0.6–0.8 mm, base cuneate, apex acuminate to acute, margin ciliolate; ***median leaves*** slightly asymmetrical, conspicuously carinate, those on main stem slightly larger than those on branches, broadly ovate, 1.5–2.5 × 0.7–1.0 mm, base subtruncate to cordate, margin ciliolate, apex long aristate, with arista up to 0.6 mm long; ***lateral leaves*** asymmetrical, those on main stem larger than those on branches, nearly oblong, 2.0–4.0 × 1–1.4 mm, acroscopic base rounded, slightly overlapping stems and branches, basiscopic base cuneate to truncate, apex acute or rounded; acroscopic margin densely long ciliolate at base and denticulate to apex, with cilia up to 0.4 mm long; basiscopic margin subentire, with sparsely denticulate at apex; ***strobili*** solitary or in a pair, terminal on the branches, compact, dorsiventrally complanate, 7.0–10.0 × 2.5–4 mm; sporophylls strongly dimorphic, resupinate; ***dorsal sporophylls*** oblong, base rounded, apex acute, margin ciliolate in basal part, other portion denticulate, with sporophyll-pteryx incomplete and ciliolate; ***ventral sporophylls*** ovate-lanceolate, apex long acuminate, margin ciliolate; ***megaspores*** pale yellow, subglobose, proximal surfaces densely verrucate, distal surfaces irregularly vermiculate to interconnected verrucate, both surfaces covered with densely echinate; ***microspores*** orange-red, tetrahedral-globose, verrucate, covered with honeycomb-like microsculpture.

##### Distribution and habitat.

China (Guangxi, Guizhou, Yunnan, Sichuan and Xizang), Myanmar, Thailand; growing on ground or rocks in forests at 100–1800 m elev. The Guizhou population was found on wet rocks in montane evergreen broad-leaved forest at ca. 1000 m elev.

##### Notes.

Within China, *S. amblyphylla* was previously recorded in Guangxi, Yunnan, Sichuan and Xizang (Zhang et al. 2013). The population (voucher *D6632*) discovered in Xingyi, southern Guizhou represents the first record of this species in Guizhou Province, although our molecular data indicate it represents a cryptic lineage. Morphologically, this species is characterised by its sterile leaves being long-ciliate at the margin and the lateral leaves oblong with rounded apices (Fig. 5). It is similar to *S. spinulosovena*, but distinctly differs by its leaves lacking a white margin (vs. obviously white margin in *S. spinulosovena*). The phylogenetic analysis indicates this cryptic species to be sister to *S. kurzii*. Morphologically, it differs from the latter in having oblong (vs. ovate-triangular) lateral leaves and broadly ovate (vs. ovate or ovate-elliptic) median leaves.

##### Voucher specimen from Guizhou, China.

• Maling River Canyon, Xingyi, 25°08'04"N, 104°57'17"E, elev. 1038 m, 2 August 2024, *S.Y.Dong, J.K.Huang & X.J.Li D6632* (IBSC).

#### Selaginella parachrysocaulos

Taxon classificationPlantaeSelaginellalesSelaginellaceae

, a new record to Guizhou, China

FFE6BC47-1BFD-5E4D-94D6-9310C6160CCC

Fig. 7

Selaginella parachrysocaulos M.H.Zhang & X.C.Zhang, Taxon 71: 1167. 2022.

##### Type.

China • Sichuan: Miyi County, elev. 1600–1800 m, in forest, 10 September 2002, *X.C.Zhang 2427* (holotype, PE, barcode 02347433).

**Figure 7.**
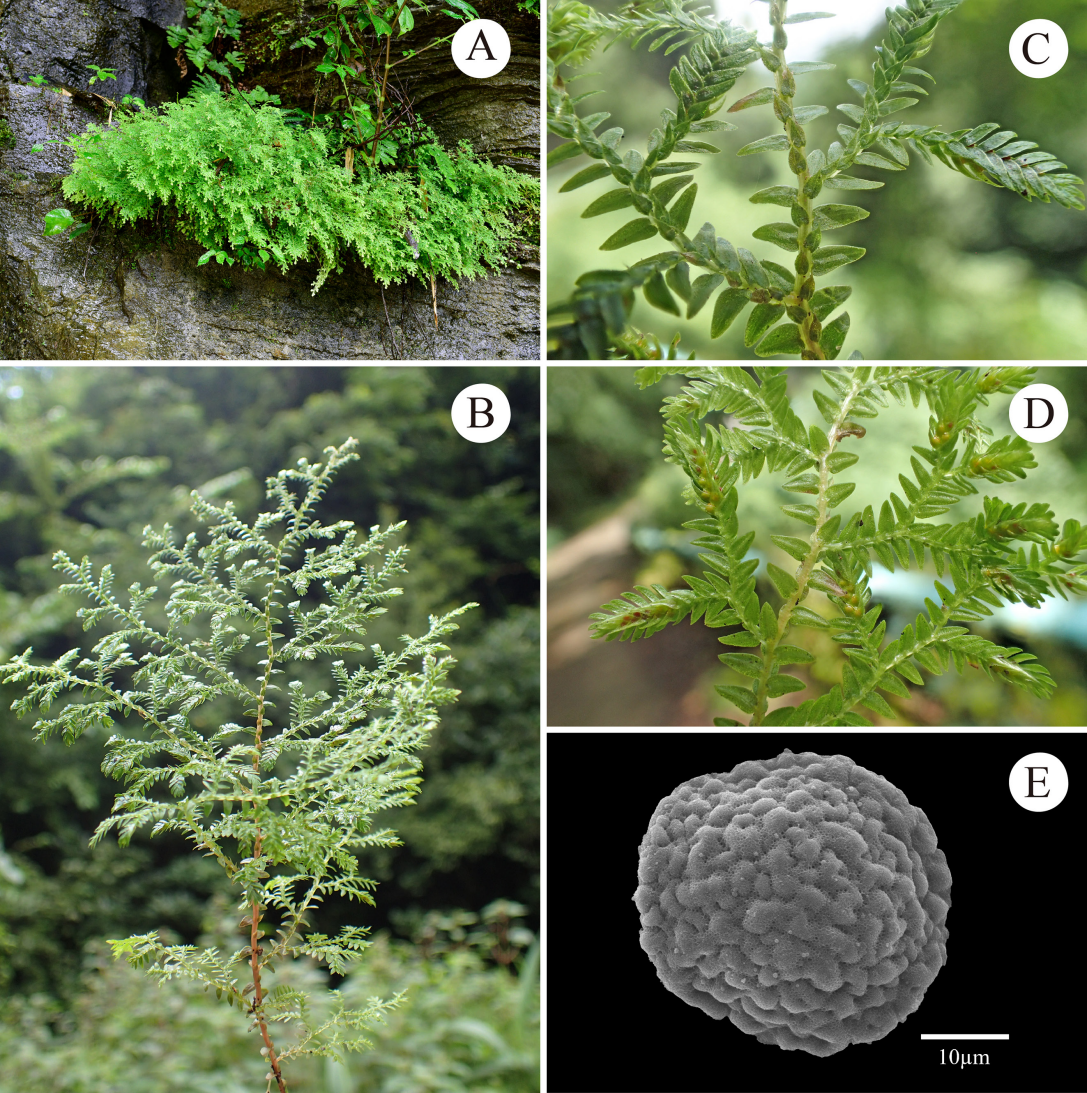
Morphology of *Selaginella parachrysocaulos*. **A**. Habit; **B**–**D**. Portion of a primary branch with secondary branches (B, C: upper view; D: lower view); **E**. A microspore (all from *D6641*).

##### Description.

***Plant*** erect, 7–30 cm, with tuber and stolon at base, tuber covered by colourless scale-like leaves; ***rhizophores*** restricted to base of main stem; ***main stem*** branched from middle or lower part, terete, glabrous, stramineous, 0.6–1.3 mm in diam. at basal part, 4.0–8.0 mm wide including leaves; ***primary branches*** alternate, 5–10 pairs, 0.5–1.8 cm apart along the main stem, once or twice pinnately branched, with ultimate branches including leaves being 3.0–5.0 mm wide; ***vegetative leaves*** herbaceous or slightly membranous, dimorphic, arranged in four rows, with obvious white margin; ***axillary leaves*** symmetrical, ovate-lanceolate or narrowly triangular, 2.0–2.6 × 0.7–1.2 mm, base rounded, apex acuminate, margin denticulate to shortly ciliolate; ***median leaves*** slightly asymmetrical, ovate, 0.8–1.4 × 0.4–0.8 mm, base cordate, 1.6–2.8 × 0.6–1.5 mm, apex aristate, margin shortly ciliolate; ***lateral leaves*** asymmetrical, ovate-lanceolate, 1.8–3.2 × 0.8–1.8 mm, base rounded, with acroscopic base slightly enlarged, acroscopic margin shortly ciliolate at base and denticulate towards apex, acroscopic margin denticulate; ***strobili*** solitary or pairs, terminal on the branches, compact, dorsiventrally complanate, 3.3–5.8 × 2.3–3.4 mm; sporophylls strongly dimorphic, resupinate, obvious white-margined; ***dorsal sporophylls*** ovate-lanceolate, with sporophyll-pteryx complete and shortly ciliolate, 1.6–2.1 × 0.4–0.6 mm, base rounded, apex acute, margin denticulate to shortly ciliolate; ***ventral sporophylls*** ovate-lanceolate, slightly carinate, 1.4–1.9 × 0.4–0.8 mm, bases rounded, apex long acuminate, margin denticulate to shortly ciliolate; ***megaspores*** yellowish, subglobose, both proximal and distal surfaces verrucate, surfaces densely covered with fine spines; ***microspores*** orange, tetrahedral-globose, surface verrucate, covered with honeycomb-like microsculpture on the surfaces.

##### Distribution and habitat.

Endemic to south-western China (Guizhou, Sichuan, Yunnan); growing on ground or rocks in forests at 1600–3100 m elev. The Guizhou population was found on wet cliffs in montane evergreen broad-leaved forest at 1855 m elev.

##### Notes.

The collection *D6641* documents the first record of *S. parachrysocaulos* in Guizhou, China. This species is morphologically similar to *S. chrysocaulos*, but differs by the slightly enlarged acroscopic base of lateral leaves (vs. strongly enlarged) and the honeycomb-like sculpture on microspore surface (vs. smooth in *S. chrysocaulos*) (Zhang and Zhang 2022). Notably, during examination of spore morphology in specimen *D6641*, we observed that the microspore surface sculpture is variable. In some microspores, a distinct honeycomb-like pattern is evident, whereas in more well-developed microspores, it appears obscure or only partially visible. This suggests that the honeycomb-like sculpture may gradually diminish as the spores reach full maturity in *S. parachrysocaulos*.

##### Voucher specimen from Guizhou, China.

• Qishe, Xingyi, 24°57'09"N, 104°45'10"E, elev. 1855 m, 3 August 2024, *S.Y.Dong, J.K.Huang & X.J.Li D6641* (IBSC).

#### Selaginella qingchengshanensis

Taxon classificationPlantaeSelaginellalesSelaginellaceae

(cryptic species), a new record to Guizhou, China

2C6D904B-0AF5-5448-A161-C6040FF550EB

Fig. 8

Selaginella qingchengshanensis Li Bing Zhang & X.M.Zhou, Phytotaxa 522(4): 286. 2021.

##### Type.

China • Sichuan: Dujiangyan City, Qingcheng Mountain, Qingchengshan Town, Wudongtian; 30°54'28"N, 103°34'6"E; elev. 960 m; 23 August 2020; *Z.L. Liang & X. Pu 085* (holotype PYU).

**Figure 8.**
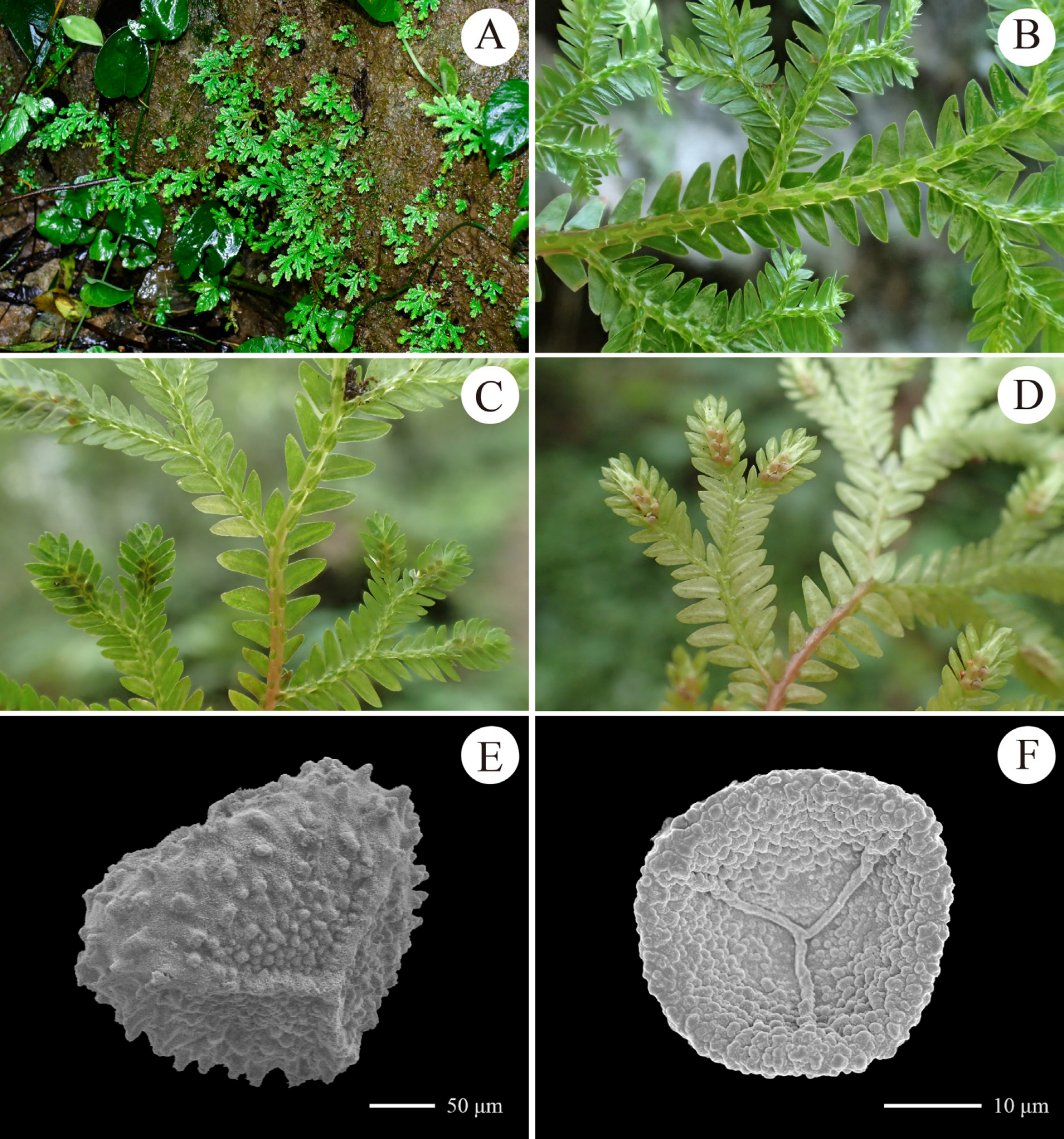
Morphology of *Selaginella qingchengshanensis* (cryptic species). **A**. Habit; **B**. Middle portion of main stem with branches (upper view); **C**. Upper portion of main stem with branches (upper view); **D**. Three strobili terminal on a lateral branch (lower view); **E**. Proximal surface of megaspore; **F**. Proximal surface of microspore (A, B from *D6664*, C–F from *D6661*).

##### Description.

***Plants*** terrestrial, suberect or ascending from decumbent base, 10–25 cm; ***rhizophores*** restricted to lower part of stem, borne on lateral side in axils of branches; ***main stem*** branched from near base, terete, glabrous, reddish at lower and middle part, while stramineous at apex, 0.8–1.5 mm in diam., 5.0–9.0 mm wide including leaves; ***primary branche*s** alternate, 5–10 pairs, 0.6–1.5 cm apart along the main stem, once or twice pinnately branched, with ultimate branches including leaves being 4.0–6.0 mm wide; ***vegetative leaves*** herbaceous, dimorphic, arranged in four rows; ***axillary leaves*** symmetrical or slightly asymmetrical, broadly ovate, 2.2–2.7 × 1.3–1.8 mm, base rounded, apex acute, margin denticulate to shortly ciliolate; ***median leaves*** asymmetrical, ovate, slightly carinate, 0.8–1.8 × 0.5–0.8 mm, base obliquely subcordate, apex long aristate, slightly reflexed, margins denticulate; ***lateral leaves*** asymmetrical, ovate to oblong-ovate, 2.8–3.7 × 1.2–1.6 mm, apex acute to obtuse; basiscopic base rounded, margin denticulate to subentire; acroscopic base rounded, slightly enlarged, overlapping stems and branches, margin denticulate to shortly ciliolate; ***strobili*** solitary or pairs, terminal on the branches, compact, dorsiventrally complanate, 3.6–6.2 × 1.6–2.5 mm; sporophylls strongly dimorphic, resupinate; ***dorsal sporophylls*** ovate-lanceolate, with sporophyll-pteryx slightly incomplete and denticulate, 1.6–1.9 × 0.4–0.6 mm, margin denticulate to shortly ciliolate; ***ventral sporophylls*** ovate, carinate, 1.1–1.4 × 0.3–0.6 mm, margin denticulate to shortly ciliolate, apex long aristate; ***megaspores*** pinkish, subglobose, both proximal and distal surfaces verrucate or papillate, surfaces densely covered with fine spines; ***microspores*** orange, tetrahedral-globose, surface verrucate, densely covered with spines.

##### Distribution and habitat.

Endemic to south-western China (Guizhou, Sichuan); growing on ground or rocks in forests at 850–1200 m elev. The two Guizhou populations were found on wet cliffs in montane evergreen broad-leaved forest at 950–1200 m elev.

##### Notes.

*Selaginella qingchengshanensis* is currently known only from its type locality, Qingchengshan in Sichuan, south-western China (He et al. 2021). The collections from Guizhou (*D6661*, *D6664*) represent the first record of this species outside its type locality. Morphologically, it resembles *S. monospora*, but differs in the reddish colouration (vs. green in *S. monospora*) of the middle and lower portions of the main stem, long-aristate (vs. aristate or cuspidate) median leaf apex and pinkish (vs. white) megaspores. *Selaginella qingchengshanensis* is also similar to *S. amblyphylla*, but distinguished by its vegetative leaves denticulate at margins (vs. long ciliate in *S. amblyphylla*).

##### Voucher specimens from Guizhou, China.

• Pogang Nature Reserve, Xingyi, 25°06'29"N, 105°05'38"E, elev. 1200 m, 5 August 2024, *S.Y.Dong, J.K.Huang & X.J.Li D6661* (IBSC); • Maling River Canyon, Xingyi, 25°08'29"N, 104°57'12"E, elev. 955 m, 6 August 2024, *S.Y.Dong, J.K.Huang & X.J.Li D6664* (IBSC).

## Supplementary Material

XML Treatment for Selaginella dushanensis

XML Treatment for Selaginella amblyphylla

XML Treatment for Selaginella parachrysocaulos

XML Treatment for Selaginella qingchengshanensis

## Additional information

### Artificial Intelligence (AI) use

The authors accept full responsibility for the content of the manuscript, including the disclosure of any use of AI.

No AI tools were used in the preparation of this manuscript.

### Author ORCIDs

J.-K. Huang https://orcid.org/0009-0003-1497-8173

Z.-Y. Guo https://orcid.org/0009-0000-3336-0713

C.-Y. Deng https://orcid.org/0000-0001-5407-5292

S.-Y. Dong https://orcid.org/0000-0002-8449-7856

### Data availability

All data are available within the paper. New nucleic acid sequences are deposited into GenBank.

## Supplementary materials

Supplementary material 1 Specimen information and GenBank accession numbers Data type xlsx Explanation note Specimen information and GenBank accession numbers of three plastid regions used in this study. https://binary.pensoft.net/file/1613243This dataset is made available under the Open Database License (http://opendatacommons.org/licenses/odbl/1.0/). The Open Database License (ODbL) is a license agreement intended to allow users to freely share, modify, and use this Dataset while maintaining this same freedom for others, provided that the original source and author(s) are credited. Jun-Kai Huang, Zhi-You Guo, Chao-Yi Deng, Shi-Yong Dong

Supplementary material 2 Specimen information and the source of plastid CDS Data type xlsx Explanation note Specimen information and the source of plastid CDS (coding DNA sequences) analyzed in this study. https://binary.pensoft.net/file/1613244This dataset is made available under the Open Database License (http://opendatacommons.org/licenses/odbl/1.0/). The Open Database License (ODbL) is a license agreement intended to allow users to freely share, modify, and use this Dataset while maintaining this same freedom for others, provided that the original source and author(s) are credited. Jun-Kai Huang, Zhi-You Guo, Chao-Yi Deng, Shi-Yong Dong

Supplementary material 3 Dataset of 37 concatenated plastid CDS Data type fasta Explanation note Dataset of 37 concatenated plastid CDS (coding DNA sequence) used in this study. https://binary.pensoft.net/file/1613245This dataset is made available under the Open Database License (http://opendatacommons.org/licenses/odbl/1.0/). The Open Database License (ODbL) is a license agreement intended to allow users to freely share, modify, and use this Dataset while maintaining this same freedom for others, provided that the original source and author(s) are credited. Jun-Kai Huang, Zhi-You Guo, Chao-Yi Deng, Shi-Yong Dong
